# Editorial: Reelin-Related Neurological Disorders and Animal Models

**DOI:** 10.3389/fncel.2016.00299

**Published:** 2017-01-05

**Authors:** Gabriella D'Arcangelo, Laura Lossi, Adalberto Merighi

**Affiliations:** ^1^Department of Cell Biology and Neuroscience, Rutgers, The State University of New JerseyPiscataway, NJ, USA; ^2^Laboratory of Neuroscience, Department of Veterinary Sciences, University of TurinTurin, Italy

**Keywords:** brain, development, neuron, reeler, autism, schizophrenia

Homozygous, loss-of-function mutations in the mouse *Reelin* gene cause a severe neurological phenotype known as *reeler*. These mutant mice exhibit marked cerebellar hypoplasia, dyslamination of cortical and hippocampal cellular layers, and malposition of specific neuronal populations in the brain stem and spinal cord. Similarly, homozygous mutations in the very conserved human *REELIN* gene cause a severe neurodevelopmental disorder known as lissencephaly with cerebellar hypoplasia (LCH). These structural defects underscore the essential role that Reelin plays in the control of neuronal migration in the prenatal and early postnatal brain. However, the Reelin protein also affects synapse formation and function in the postnatal and adult brain, and this function is reflected in the manifestation of behavioral and cognitive deficits in heterozygous *reeler* mice that express reduced levels of Reelin and do not exhibit overt brain structural defects. A reduction in REELIN expression is also found in human patients affected by neuropsychiatric disorders, including autism, schizophrenia, and depression. How does a deficit in REELIN expression contribute to these diseases? In order to understand the potential role of Reelin dysfunction in neuropsychiatric disorders we need to gain a deeper understanding of the molecular mechanisms by which this protein controls all aspects of brain development and function. This Research Topic is a collection of reviews that summarize and interpret many recent findings in the Reelin field. The Topic also includes original research articles that provide novel information on Reelin expression and function in different regions of the central nervous system.

Reelin is a large glycoprotein that is secreted by different neuronal populations at different stages of brain development. Shortly after its discovery, it became clear that Reelin is subject to proteolytic cleavage after secretion, resulting in the generation of multiple extracellular protein fragments. The functional significance of this process was not well understood. In recent years, several studies have shed some light on this event, identifying specific cleavage sites, and addressing the consequence of proteolytic cleavage for Reelin biological function. Ranaivoson et al. review structural aspects of Reelin as a ligand, discussing its proteolytic fragments, and the binding of uncleaved and cleaved products to known cell surface receptors. Lussier et al. further review Reelin proteolytic cleavage and its potential role in modulating synapse function in the normal adult brain or in neurodegeneration. Two reviews then discuss in depth molecular aspects of Reelin signal transduction that are initiated by distinct Reelin protein isoforms. Lee and D'Arcangelo discuss how different Reelin ligands trigger different signaling pathways, and thus control different functions such as neuronal migration, maturation, and synaptic activity. Bock and May further review in depth canonical and non-canonical signaling pathways, emphasizing the role of different pathways in the control of the neuronal cytoskeleton. Together, these reviews provide a detailed and comprehensive summary of the current state of the field's mechanistic understanding of Reelin signaling activities.

In the prenatal forebrain Reelin is expressed mainly by Cajal-Retzius cells in the marginal zone to control the radial migration of excitatory neurons, whereas a subpopulations of interneurons mainly express this protein in the postnatal forebrain likely to modulate synaptic function. In the cerebellum, Reelin is mainly expressed by granule cells and granule cell precursors, controlling the radial migration of Purkinje cells and possibly affecting cerebellar circuit function. In addition, less-characterized cell types also express Reelin in other regions of the brain and spinal cord, affecting positioning and connectivity of several neuronal target populations. A series of original research articles in this Topic reveal novel aspects of Reelin expression and function in the central nervous system. Carceller et al. describes the phenotypical identity of unique Reelin-expressing cells in the piriform cortex. Bosch et al. investigated the effects of *in vivo* Reelin overexpression on the development of synaptic structures in the adult hippocampus. They demonstrate striking effects on the morphology of presynaptic as well as postsynaptic structures. Furthermore, they show that Reelin overexpression affects the trafficking of NMDA receptor subunits and associated proteins from synaptic toward extrasynaptic and cytosolic sites, thus modulating glutamatergic neurotransmission. Granule cell dispersion in the dentate gyrus is frequently associated with temporal lobe epilepsy in human patients. Using organotypic slice cultures Orcinha et al. re-examined the previously noted correlation between Reelin loss and granule cell dispersion after seizures. They show that the central fragment of Reelin rescues the abnormal migration of dentate cells, establishing a causal relationship between Reelin loss and granule cell dispersion in epilepsy. Cocito et al. focused on the cerebellum, and documented alterations in neuronal proliferation and apoptosis in the homozygous *reeler* mouse lacking Reelin. Finally, Vaswani and Blaess review findings related to the expression pattern and role of Reelin in the ventral brain stem and spinal cord. They describe specific neuronal populations that express either Reelin or components of the canonical signaling pathway, and review data in support of the notion that Reelin affects specifically the final stages of migration of its cell targets. However, unlike the neocortex, Reelin appears to regulate both tangential (midbrain and spinal cord) and radial migration (hindbrain). Future work will be needed to clarify the mechanism underlying these activities, and to determine whether, as in the forebrain, Reelin affects the maturation or synaptic function of brain stem and spinal cord target neurons.

The association and possible involvement of Reelin dysfunction in neuropsychiatric disorders is addressed by several excellent reviews. Lammert and Howell discuss human REELIN heterozygous mutations in autism spectrum disorder. They describe the several *de novo* mutations identified so far in autistic patients, their location and their potential effect on Reelin protein structure and brain development. However, the author caution that, given the lack of an autistic phenotype in heterozygous *reeler* mice or in human subjects carrying *REELIN* gene deletion, second-hit mutations, or environmental insults may be necessary to develop the disease. Guidotti et al. delve into the association between human REELIN dysfunction and schizophrenia, and review the experimental evidence from post-mortem human tissue and animal models pointing to epigenetic mechanisms that cause reduced *REELIN* gene expression in GABAergic corticolimbic neurons. The studies so far suggest that this deficit may disrupt synaptic connectivity and predispose human subjects not only to schizophrenia, but also to bipolar disorder. Caruncho et al. focus on the role of Reelin in the pathogenesis of depression, and discuss data from an animal model of the disorder, produced by repeated corticosteroid injections. This treatment causes the loss of Reelin-positive cell in the subgranular zone of the dentate gyrus, where adult neurogenesis takes place. Finally, Ishii et al. provide a thorough summary and critical discussion of the human genetic and experimental animal evidence relating Reelin dysfunction to neuropsychiatric disorders, and emphasize how knowledge of the Reelin signaling mechanisms could potentially be translated into therapeutic intervention for neuropsychiatric disorders.

Overall, the data presented in this Topic provide strong support for the idea that Reelin plays and essential role of in brain development and in adult brain function, and compel us to further investigate this protein's function using multidisciplinary approaches, including structural, cellular, anatomical, physiological, and genetic approaches, in the hope that the newly acquired knowledge will help us to develop much-needed forms of pharmacological intervention for the treatment of neuropsychiatric disorders.

## Author contributions

GD wrote the editorial. LL and AM read and approved the text.

### Conflict of interest statement

The authors declare that the research was conducted in the absence of any commercial or financial relationships that could be construed as a potential conflict of interest.

